# Including Prisoners in Research Design: Codevelopment of a Practical Guidance Toolkit to Support Intervention Delivery to Address the Physical and Mental Health of Older Prisoners (PAMHOP) Study

**DOI:** 10.1111/hex.70246

**Published:** 2025-06-09

**Authors:** Amanda E. Perry, Thirimon Moe‐Byrne, John Schofield, Lisa Ashton, Noemia Siqueria, Sarah Knowles, Prof Rachel Churchill, Tim Colman, Steve Parrott, Kevin Williamson

**Affiliations:** ^1^ Department of Health Sciences University of York York UK; ^2^ Department of Archaeology University of York York UK; ^3^ Centre for Reviews and Dissemination (CRD) University of York York UK; ^4^ National Prison Radio Association London UK; ^5^ Rotherham Doncaster and South Humber NHS Foundation Trust Doncaster UK

**Keywords:** ageing, codevelopment, common mental, cost, criminal justice, implementation, physical health

## Abstract

**Introduction:**

Over the last decade, the number of older people in custody with common mental and physical health problems has increased. Little is known about the effectiveness of interventions targeting this age group.

**Objective:**

To codevelop a practical guidance toolkit(s) to support the delivery of interventions to benefit the common mental and physical health of older people in custody.

**Methods:**

Twelve 3‐h workshops between March and April 2023 were conducted with 26 participants at two prison sites in the North of England. The six workshops in each site consisted of research‐based activities and interlinked taster sessions. The research data were collected by the research team to identify the causal links between common mental and physical health problems; activity preferences; the feasibility, acceptability and sustainability of delivering the activities and engagement barriers, which formed a bespoke questionnaire. The taster sessions (drugs and alcohol for males, chair yoga for females, books and crafting, and a historical session for both males and females) were delivered by the research team and prison staff. Feedback from the workshop participants was documented using an adapted questionnaire to record the experiences of those taking part. A micro‐costing framework was used to estimate the cost.

**Results:**

Similar common mental and physical health factors were listed by males and females. Symptoms of common mental health problems were improved by engaging with others of the same age, conducting activities outside and a consistent prison regime. Activity preferences (e.g., creative activities) were underpinned by a sense of purpose, learning new things, gaining and sharing skills. Engagement was supported by building good relationships and offering guidance through peer support, with activities led by staff of a similar age. Activities were more likely to be deemed feasible, acceptable and sustainable when aligned with the prison strategy and in conjunction with the regime. The average cost per participant for the intervention delivery was higher for males than females (£157 vs. £89).

**Conclusion:**

Older people in custody report high levels of mental and physical health problems. Engagement with people in custody helps to support the development of interventions maximising possible health benefits. Further research is required to develop an evidence‐base for this group of people in custody.

**Patient or Public Contribution:**

People in custody were involved in the design and implementation of the workshops. The Project Advisory Group advised us on our research methodology and evaluated the feasibility, acceptability and sustainability of the activities using a questionnaire; they also provided practical advice about the project delivery.

## 1 Introduction

In the last two decades, the worldwide population of incarcerated older (aged 50 years and above) people in custody has risen disproportionately to those that are younger [1, 2]. In the United Kingdom, this represents a 159% increase for individuals aged 50–59 and an increase of 243% for those aged 60 and above [3]. Those that are ageing in custody experience the onset of illness between 10 and 15 years earlier than the rest of the population and are considered appropriate for geriatric measures of care [4, 5]. Such accelerated ageing is attributed to lifestyle choices, social deprivation and the effects of incarceration itself [6]. The process of delivering healthcare is exacerbated by rising costs (£119 billion in 2019) [7], poor access to healthcare in the community and high levels of social exclusion for those that are released [8], with a high proportion of those released reporting at least one moderate or severe health condition [9].

The most prevalent conditions include common mental health disorders (e.g., depression and anxiety) and physical health conditions such as diabetes, long‐term pain and respiratory disease such as Chronic Obstructive Pulmonary Disorder (COPD) [10]. Gender disparities and health inequalities exist; with females more likely to report poorer health compared to their male counterparts. Such differences support the requirement to tailor interventions based on the gendered needs of those receiving them [11, 12].

Although innovative codevelopment methods in other areas of health and social care research include patients and carers in the design and intervention adaptation process [13, 14, 15], the engagement of people in custody has been described as under‐developed [16] with participants not fully acknowledged in the process [17] and codevelopment engagement rated as poor [18, 19]. A lack of service user engagement hinders the acceptability and feasibility of interventions leading to problems of adherence [20, 21] and high levels of attrition [22, 23]. Additionally, given the constraints around resources in prisons [24, 25] having a good understanding of the cost and cost‐effectiveness of intervention delivery is an important consideration.

Meta‐analytical studies of adherence in the community suggest that it is affected by the intervention ‘place’, ‘delivery’ and ‘type of diagnosis’ [20, 21]. Other research has shown that interventions are more likely to be deemed acceptable (i.e., is there widespread appeal for this activity?), feasible (i.e., how, who and where can the intervention be delivered?) and sustainable (i.e., what resources are required?) when service user engagement forms part of the development process [25]. Additionally, the voice of this service user is often not heard [26]. Given the recognised benefits of engaging with service users in the intervention development process, the objective of this paper was to develop a practical guidance toolkit(s) to support the delivery of interventions for older people in custody with common mental and physical health problems. This was co‐produced using evidence from a systematic review (Phase 1), a prison site survey, interviews with people in custody and prison staff (Phase 2). Both phases formed part of a larger study referred to as PAMHOP (Physical And Mental Health of Older Prisoners) (https://www.york.ac.uk/healthsciences/research/mental-health/projects/physical-mental-health-older-prisoners/) and are published elsewhere [27, 28]. This paper provides a brief description of the prior two phases of the project and reports on Phase 3, a series of workshops aimed at producing the practical guidance toolkit(s) for older people in custody.

## 2 Setting and Prior Development Work

Participants for Phases 2 and 3 of the study were recruited from two prisons in the North of England with up to 1300 people in a male Category C prison with up to 900 men and a female prison with up to 350 women.

### 2.1 Systematic Review Evidence (Phase 1)

Phase 1 of the PAMHOP study included a systematic review (PROSPERO ID CRD42021281384) to identify randomised controlled trials (RCTs) solely targeting the effectiveness of interventions to improve the common mental health of older people who were 50 years and above in custody. No studies targeting this age group were identified. A proportion of studies (six containing men [29, 30, 31, 32, 33, 34] and two containing women [35, 36]) reported ‘within study samples’ of people aged 50 years and above and representing less than 10% of the total study sample size. These studies reported common mental health outcomes for anxiety and depression, but no single study reported any physical health outcome measures relating to either COPD, diabetes or obesity. Given the limited knowledge, we were unable to comment on the effectiveness of interventions for this age group. Nevertheless, the TIDieR checklist was used to identify facilitators and barriers to implementation [37]. We found that overall, levels of acceptability and feasibility were not consistently reported across the studies. There was little evidence reported on ‘how’ we implement and ‘deliver’ these interventions; few reported any adaptations nor measured fidelity. Studies did report varying degrees of attrition (ranging from 0% to 61%) supporting the wider literature which reports on the challenges of intervention implementation and engagement within this setting [38].

### 2.2 Surveys and Interviews (Phase 2)

Data in Phase 2 were collected between July 2022 and January 2023 [28] and consisted of (i) a survey of people in custody aged 50 years and older (in the same two prison sites) and (ii) a series of semi‐structured interviews with those who reported a physical illness (either obesity and or diabetes and or COPD) and scored above 10 on measures of depression using the Patient Health Questionnaire‐9 (PHQ‐9) [39] and/or anxiety using the Generalized Anxiety Disorder‐7 (GAD‐7) [40] on the survey. Interviews with people in custody and prison staff were conducted to understand more about the needs and activities of older people in custody; activities to support common mental health problems; the practical and logistical elements required to sustain an activity; key skills required for people upon release and barriers to accessing healthcare within prison. These topics were further explored during virtual consultations with our Project Advisory Team who provided further contextual information^1^ and represented each prison site and contributed to Phases 2 and 3 of the project. Members of the team were recruited voluntarily specifically to advise on the project and were not paid for their involvement in the study.

## 3 Methods

### 3.1 Prisoner Workshop Recruitment (Phase 3)

**Figure 1:**
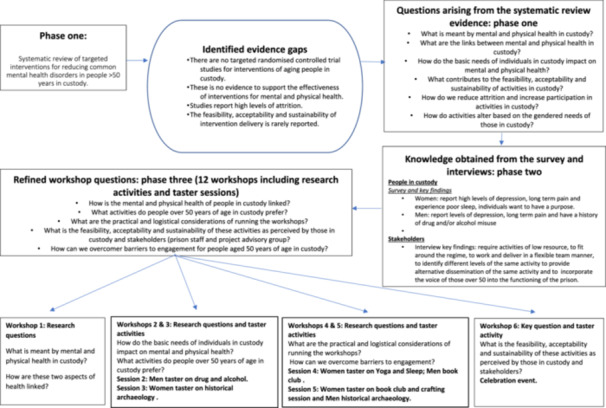
Project overview.

Between 6 February and 21 March 2023, those in custody (and already agreeing to take part in Phases 1 and 2 of the study) were individually approached by the CI AP to identify their willingness to take part in the workshops (Phase 3). Workshops (lasting up to 3 h) were conducted once a week in each site for a period of 6 weeks. The workshop principles were designed around the Pearson‐based approach to intervention development [41] and consisted of activities generated from the research gaps in Phases 1 and 2 (Figure 1). Those attending three or more workshops received a £10 prison account reimbursement. Prison staff who supported the delivery of the workshops and the Project Advisory Team [^1^] provided advice and gave their informed consent to evaluate the feasibility, acceptability and sustainability of the activities using a specifically designed Qualtrics questionnaire [42]. All data were anonymised. Ethics were approved by the East of England‐Essex Research Ethics Committee (REC reference: 22/EE/0120), the HMPPS National Research Committee (NRC), each prison Governor and Health care provider.

#### 3.1.1 Overall Workshop Principles and Structure

Figure 1 shows the project phases and identified evidence gaps from the different phases of the study: Phase 1 (the systematic review) [27] and Phase 2 (the survey and interviews) [28]. The questions were further refined and used to guide the workshop structure and the content of each workshop (Phase 3).

The results from Phase 2 identified activity requests suggesting that no‐one activity was going to appeal to the workshop participants. The findings did support group activities providing people with the opportunity to socialise with people of their own age and recognising a sense of achievement. The final choice resulted in a combination of research activities based upon the known evidence gaps and a series of taster activities based on the following: (i) what participants had told us they would like to do and the results from the survey and interviews in Phase 2, (ii) the willingness of staff to offer an activity and (iii) the expertise of the research team. The research team were keen to promote prison staff's ‘ownership of the activities’ on the premise that the activity may be sustained beyond the life of the research project. The ownership promotion supported at the time the prison business strategy; of which one element was an aspiration to provide ‘more on wing and in cell activities based on the talents of their own prison staff’.

Each prison site conducting one health‐related activity (for men a drug and alcohol rehabilitation session, and for women a session on mindfulness and chair yoga to enhance sleep), a book club and puzzle or crafting session (Session 4 for the men and Session 5 for the women) and a historical session (Session 3 for the women and Session 5 for the men).

To support the logistical requirements of the session, delivery sessions were held on the ‘line route’ movement and were attended by at least one operational prison staff member. Lastly, evidence from the systematic review literature [38] and other research [26] supported the idea that older people in custody experienced ‘a lack of voice’ in the prison system. For this reason, the workshop Sessions 2–4 in the male prison and Sessions 3–5 in the female prison) were attended by the Prison Radio Association. The Prison Radio Association disseminated and broadcast the voices nationally across the prison estate producing a five‐part series referred to as ‘50 plus’.

#### 3.1.2 Workshop Content

Workshop 1 used prior knowledge from a Theory of Change (ToC) model developed by Perry et al. [43] to examine the causal factors of mental health of men in custody. The purpose of the workshop was (i) to interrogate the existing model to identify differences by gender and (ii) to include the causal constructs of physical health and the comorbidity between mental and physical health. Mental and physical health conditions were limited only to those outcomes that were sought in the prior systematic review and survey focusing on the commonly reported mental health conditions of depression and anxiety and the physical health conditions of obesity, diabetes, COPD and long‐term pain (Supporting Materials 1). The information was used to enhance an emerging logic model that had been produced from the evidence collated from the interviews (Supporting Materials 2).

Workshops 2 and 3 consisted of three elements. The first addressed the issue of ‘need’, the second involved an activity to identify activity preferences and the third included different taster activities. The first element reviewed the key findings of the survey to the group and talked about the concept of ‘need’ and second, how that need impacted their own mental and physical health. It promoted the idea that activities were more likely to have a beneficial impact if they targeted the identified needs of the group. The recorded needs were differentiated to identify any gender differences.

The second element presented the activities gathered from the survey and interview material. In this activity, the group were asked to create a hierarchical list based on activity preference and then activity preference based on the perceived benefit to mental health. The preferences were ranked using a method like the James Lind Alliance method of prioritisation (https://www.jla.nihr.ac.uk/jla-guidebook/). Once ranked, any that fell outside of the top 10 were removed from the list. The resulting list of activities formed a questionnaire that was used to assess the feasibility, acceptability and sustainability of each activity by those in custody, prison staff and the Project Advisory Team in Workshop 6.

A combination of different taster sessions was offered across these two workshops. Participants in the male prison received a session led by prison staff from the Drug and Alcohol Rehabilitation Service (DARS). The workshop involved a brainstorming activity to consider what is meant by ‘Addiction’ and what we mean by ‘Recovery’. This promoted an insight into the different journeys the group had experienced through drug services and how drug use had changed as they became older. The workshop discussions were recorded by the PRA for the purpose of the radio broadcast. The discussion was written verbatim, and the narrative transcription was used to identify the key elements or ‘magic ingredients’ that contributed to positive recovery. Examples of these ‘magic ingredients’ were listed as potential mechanisms of change and included positive family connections, gaining family trust, having a pride in yourself, building up a new social community opportunity, having a belonging to something, relating to people of their own age, having a safe environment, using peer to peer support and having positive inspirational role models from older ex‐addicts that have been successful.

For Session 3 (the women) and Session 5 (the men) experienced a workshop that included a historical narrative and involved two tasks: a map regression exercise (undertaken within a classroom) and the drawing of a building elevation (out of doors). Different maps over the passage of time were used to examine changes in the presence of buildings, changes in landscape and the location of prison buildings. The drawing of the building elevation was conducted in smaller groups where pairs were shown how the scaled drawing could fit within the page including marking the footings, gutters, windows, door frames and brick work and marking of air bricks. The groups reflected on the impact of having tried something that they had not done before. This helped to build an unexpected sense of confidence. It helped to generate a sense of place and belonging of ‘knowing where you are’. It provided an opportunity for people to work together outside as a team and provided a different perspective to the prison environment that sought to widen the interests of the group. As a final element of the session, the drawings were scanned into one document, linked and then framed for each participant as a remembrance of the activity and provided and recognise a sense of achievement of all those taking part and presented in Workshop 6 (for more detail see [44, 45, 46, 47]).

Workshops 4 and 5 explored the issue of engagement using character persona using three male and three female previously co‐produced (prison‐based) character persona [48]. Engagement, acceptability and high levels of attrition in the delivery of prison‐based interventions were noted as a key barrier in the findings of the systematic review and interview data. We asked participants to identify how to overcome the barriers each person for each character was asked to consider (i) what would encourage (character name) to do an activity? And (ii) what kind of activities might benefit (character) particularly in relation to mental and physical health to produce a solution‐focused approach to the barriers that had been previously presented in the interviews (e.g., age; ethnicity: Supporting Materials 3). The characters were adapted to incorporate evidence collected from the survey and interviews in Phase 2 of the project and were drawn from a diverse and inclusive approach by considering different nationalities, ethnicity, age, criminal background and health needs. This ensured that the character personas were relatable.

The accompanying taster sessions included an adapted chair yoga and sleep activity (Session 4) and a book and crafting activity (Session 5) for the women, and a book club and puzzle (Session 4) and the historical session reported above (Session 5) for the men. For the adapted chair yoga session, two members of the prison mental healthcare team delivered the session. It involved exercises to recognise the breath, a gentle warm up and stretch routine and a relaxation script to promote sleep. The session promoted sleep (something that was identified as a need for this group) and led into a general discussion about how existing gym sessions could be adapted to accommodate only those that were 50+ by making them shorter and including quieter music. For the book club and craft sessions, the women made a list of four books and voted on the book of choice to read. The books were ordered from a charity (https://giveabook.org.uk/project/prison-reading-groups/who) sent 18 (only 8 were requested) copies of the book. The session was held in the prison library with two staff escorting. Once in the library, the group had a lively informed debate and discussion about the book, its characters, how perceptions of the character had changed their own views and personal experiences. During the library session, some women who had not attended before were given the opportunity to choose other books and read.

The second part of the session was a crafting activity led by the women themselves who wanted to share, teach and demonstrate their own crafts to others in the group. Two members of the group taught others how to make an origami butterfly and demonstrated paper craft. In the male prison, the book club session emerged differently as no‐one had received the books in advance of the session. Instead, we discussed with the group how the session could be delivered within the restraints of prison resources. The library offers a wide range of other facilities including access to computers to complete a job search, providing desks and is akin to a space you might find in the community. They discussed opportunities for a creative writing group with a competition element with a small reward (e.g., shampoo or a dictionary). They presented ideas to generate a pick of the month list, including displays in the library of short book reviews, access to coffee and boardgames. In the absence of being able to facilitate access in advance of the book club session, alternative ideas about a debating group using access to readily available prison newspapers and magazines (perhaps considered a short read) could provide an opportunity for current debate. The benefits of the debating group included having an opportunity to hear other people's views, talking to others of the same age, respecting others’ views, listening, keeping abreast of current changes, improving knowledge, skills and confidence. It was thought that the member of staff with the group could participate to enhance prisoner–staff relationships in the prison. The discussion progressed into engagement with prisoners that were younger than themselves to share learning and encourage use of the library. The group were keen that any books obtained from charities could be donated elsewhere. They spoke of donating books from the club to schools or offering the books in an exchange process (e.g., £1 per book for other people in custody to buy). The group saw this as a way of raising funds that could generate a self‐sustaining approach to purchasing new books. Some members of the group spoke passionately about how books that were donated to schools could upon release be invited to visit the school to provide an inspirational speech. These ideas were fundamentally linked to having a need for a sense of recognition and or achievement for what they had done.

Workshop 6 was used to evaluate the feasibility, acceptability and sustainability of the activity preference listed that were generated in Workshop 2 and provide a final sense of achievement from the sessions. The activity preferences were compiled into a questionnaire using a Qualtrics survey tool [42]. Prison staff and the Project Advisory Team were emailed and asked to complete a questionnaire via a hyperlink. Workshop participants completed the same questionnaire via pen and paper. For each activity participants were asked to consider the feasibility of the activity location (outside, in cell, online, on wing, in education, gym and library), acceptability (who should be able to access the activity) and the sustainability of the delivery mode (i.e., should the activity be delivered individually or in a group?). Activities were rated on a five‐point Likert scale (ranging from very unlikely to very likely). An accumulated overall rating by gender and stakeholder group (Supporting Materials 4). The last workshop included a ‘sense of achievement’ with certificate presentations by a member of the prison management team, refreshments and a lay person summary briefing was provided. The women they were presented with a framed copy of the building elevation drawn in Workshop 3. All participants were given an adapted feedback questionnaire (Supporting Materials 4) [49] to capture the experiences of those taking part in the workshops. The evidence generated through the workshops and in particular the ‘magic ingredients’ supported the refinement of a logic model using the GUIDED framework [50] (Supporting Materials 5).

### 3.2 The Workshop Costs

The workshop costs were collated to develop and inform a subsequent cost‐consequences analysis (CCA). The total and average cost of delivering the workshops by site was calculated by recording prospectively the resources used (staff and consumables) and multiplying them by the unit costs. We estimated the costs of planning and preparing sessions (capital costs, one‐off investment) and delivery of the training sessions (recurrent costs: personnel, attendance fee, stationery and catering). As people in custody were paid as if they were doing their usual prison job whilst attending the workshops, we also calculated the opportunity costs based on an estimate of the prisoner's income in the United Kingdom (£10 per week and £2 per training session) [51, 52]. Costs were collected in the local currency (British Pounds, 2022 prices). Personnel costs applied to the English pay scale for academic and non‐academic jobs.

## 4 Results

### 4.1 Prisoner Recruitment to the Workshops

**Figure 2:**
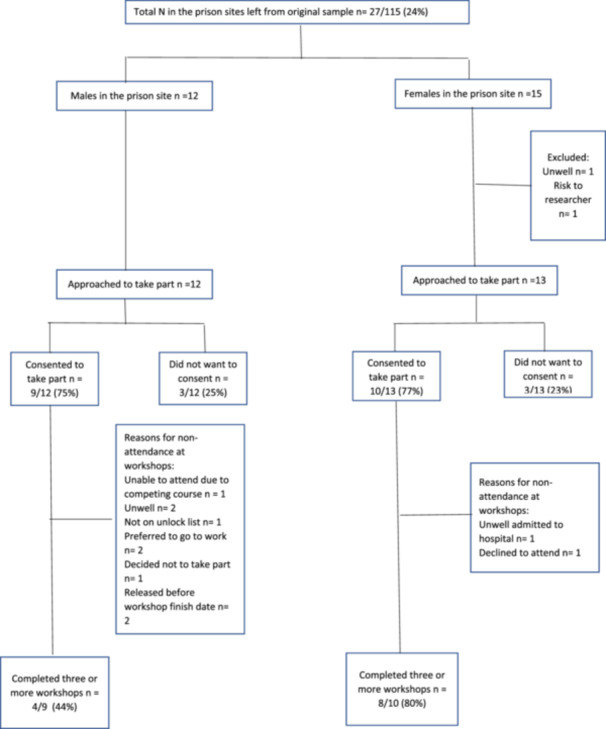
Flow diagram of participants.

Between the two prison sites, 27/115 people who had engaged in Phase 2 of the PAMHOP study remained in the prison at the time of conducting Phase 3. Of those, 25/27 were approached (12 men [9/12, 75%] and 13 women [10/13, 77%]) and agreed to take part in the workshops. Men were less likely 4/9 (44%) to attend the workshops than women 8/10 (80%) (Figure 2). For the women, one declined to attend in preference for work after the first session. Eight others attended the full 6‐week course, one person who was admitted to hospital for an operation after Session 3 and did not return to custody. Of the men, one vocal individual challenged the worth of the project and following the first session, one person said the workshops were not for him, and the other two chose to go to work. One person was unwell for the first session, and then was not on the prison unlock list for Session 2, but then subsequently attended every session. One person was released after Session 4.

### 4.2 Evidence Generated From the Research Workshop Activities

#### 4.2.1 Workshop 1: Exploring the Relationships Between ‘Common Mental’ and ‘Physical’ Health in Custody

##### 4.2.1.1 Common Mental Health

Both men and women had similar responses to what was meant by ‘common mental’ and ‘physical health’. They provided examples of how poor mental health made them feel (angry, irritated, hopeless and isolated, worthless, lonely and failure) and act (withdrawn, staying in room, want to be left alone, poor hygiene and poor sleep). Examples of how to improve mental health in custody for men included time out of cell, not sleeping during the daytime, exercise and relaxation, low caffeine, talking to others in custody, kindness and reading. For women, positive mental health was associated with having a consistent regime, faith, friendships, family time, spending time outside, having good mechanisms for communication, and demonstrating empathy for others and not being hungry.

##### 4.2.1.2 Physical Health

**Figure 3:**
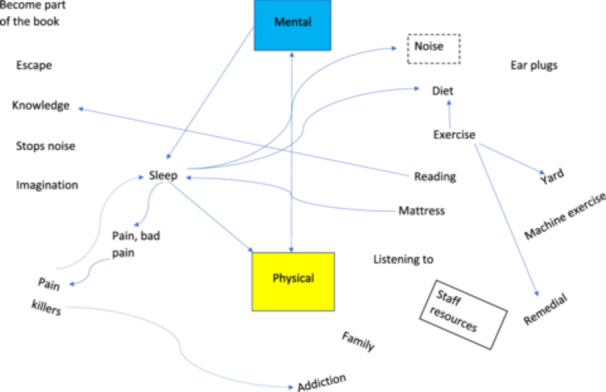
Example mapping constructs.

For men, factors associated with poor physical health included being overweight, having back pain, a lack of exercise, poor diet, poor eyesight and poor‐quality mattresses. For women, concerns about their physical health were linked to specific conditions such as sciatica, arthritis, menopause, blood pressure, memory and hearing loss, poor sleep and bladder control. Factors that improved the physical health of all participants regardless of gender were access to appropriate physical exercise, activities outside, yoga (for the women), art and craft sessions, talking to others of the same age and adapted gardening activities (Figure 3).

#### 4.2.2 Workshops 2 and 3: An Assessment of Prisoner Needs and Activity Preferences

##### 4.2.2.1 An Assessment of Prisoner Needs

The basic prison needs of those in custody and the impact this had on an individual's mental and physical health problems were similar. All participants reported the basic need for ‘purposeful activity’. This included having a job within the prison or having something to promote and/or stimulate the brain and avoid boredom and develop skills. Having a suitable prison job enabled people to have an identity, which led to improvements in self‐worth and self‐esteem. Positive perceptions of mental health and well‐being were linked to healthcare access within the prison. Individuals reported that the promotion of mental healthcare services, access to psychological services and professionals that supported their physical health requirements (e.g., a dietician) were important factors in maintaining good mental health and well‐being. Individuals discussed the requirement for physical exercise in different forms. For example, all mentioned activities relating to walking, dancing (for the women), and gardening. All recognised the need for activities to promote connectivity between other people in custody of their own age. This was aimed at reducing isolation and promoting the ability to talk to others. For those with family connections, talking on the phone, via email and having face‐to‐face visits was an important part of maintaining mental health and well‐being.

##### 4.2.2.2 Activity Preferences Rated by Prisoners, Prison Staff and the Project Advisory Group

Activity preferences were ranked with more than one activity ranking as equal by the different stakeholder groups. This resulted in 13 (reported by stakeholders in the female prison) and 19 (reported by stakeholders in the male prison) identified activities. Women were interested in recreational activities such as watching TV, listening to music, exercising, engaging in arts and crafts, cooking and socialising. Similarly, men were interested in recreational activities as well as activities involving learning such as attending short courses, workshops and getting a qualification.

#### 4.2.3 Workshops 4 and 5: The Practical and Logistical Considerations and Identified Ways to Overcome Barriers to Engagement

##### 4.2.3.1 Practical and Logistical Considerations

Prisoners and staff reported that most activities could be delivered in a variety of different places. Examples included the library, education area, on the wing or in the cell. Participants were keen to explore physical activities that could occur outside, or individually in a cell, on the wing, in the gym or online (where laptops are available). Those in custody preferred activities that were group‐based to promote opportunities to socialise and talk to people of a similar age. Who gets access to such activities was debated within the groups. Some felt that reserving incentivised activities such as extra time in the gym or dancing should be only for those in custody with an ‘enhanced’ status of living.

##### 4.2.3.2 Barriers to Overcome Engagement

Strategies to help support the engagement of people in custody to attend planned activities included building relationships within the prison community including prisoner peers, prison staff, family and chaplaincy members. Engagement was facilitated by establishing trust in a process that involved supporting each other to resolve problems and address life events before release (Table 1). Examples of trust building included the provision of peer‐to‐peer (e.g., facilitating language barriers) and being able to support and empathise with another person's situation.

**Table 1:** Adaptations of the character persona and ways to improve engagement.

Character summary	Listed additional physical health conditions	Listed additional mental health conditions and well‐being	Barriers to engagement in each persona	Responses from the male prison site: Ways to improve engagement	Responses from the female prison site: Ways to improve engagement
*Anjumann/Patrick*. Recalled on licence Poor command of English language Retired, previously a care worker Isolated in her/his cell	Overweight Suffers from bouts of depression Low self‐esteem and confidence		Language barrier Finds it hard to get on with younger prisoners Prison rules restrictive retirement age Low self‐esteem and confidence Conflict between people in custody	Good communication with peers on the wing/staff/healthcare/key workers/personal officer Seek friendship/build trust Attend well‐being clinic Learn to speak English Good diet/healthy eating/lose weight Sort his life and problems on the outside Use antidepressant medication Find a new interest and upskill	Needs to be in a group of people who are the same age Talk to a peer that can help advise her Identify another Polish prisoner who might be able to translate/talk to in her own language Identify if English can be taught Speak to her key worker Someone of her own age should introduce her to an activity
*Sofia/Joseph*. Lengthy sentence for fraud after a good career. Shocked by his/her sentence. Struggling to adjust to prison life. In debt	Experiencing physical back pain: Because of a motorbike injury. Isolated, lonely Feels unsafe and intimidated Mental health is starting to suffer Not looking after herself		Feeling unsafe in prison Budget issues In pain	Find a job/seek friendship/build trust/buddy system Manage pain/well‐being/medication Adjust to prison life, attend an anger management course Develop a good relationship with the family Talk to safer custody or over 50s wing rep	Do an activity with just a couple of people on the wing Building up an activity into a group Having a buddy to talk to Speak to chaplaincy to arrange a visit
*Nadia/Andrew*. Long history of self‐harm behaviour Committed a high‐profile crime Has restrictions on her/his movement around prison	Diabetic: Must have a controlled diet Feels out of control Anxious Is not sleeping well Reluctant to come out of her cell Social stigma of being escorted around prison		Type of crime committed Physical problem	Good communication (1 to 1), friendship/build trust, over 50s wing representative Break through the barrier of self‐harm Attend well‐being clinic, see psychologist/take medication Attend exercise classes and keep fit Engage with group activities Let him know that help is available	Elevate self‐harm behaviour Support from a listener Support from a dietician Work on a one‐to‐one basis then build up into a group Peer mentor to help monitor her food

#### 4.2.4 Workshop 6: Feasibility, Acceptability and Sustainability of Proposed Activities and Taster Activity

##### 4.2.4.1 Activity Feasibility and Acceptability

**Figure 4:**
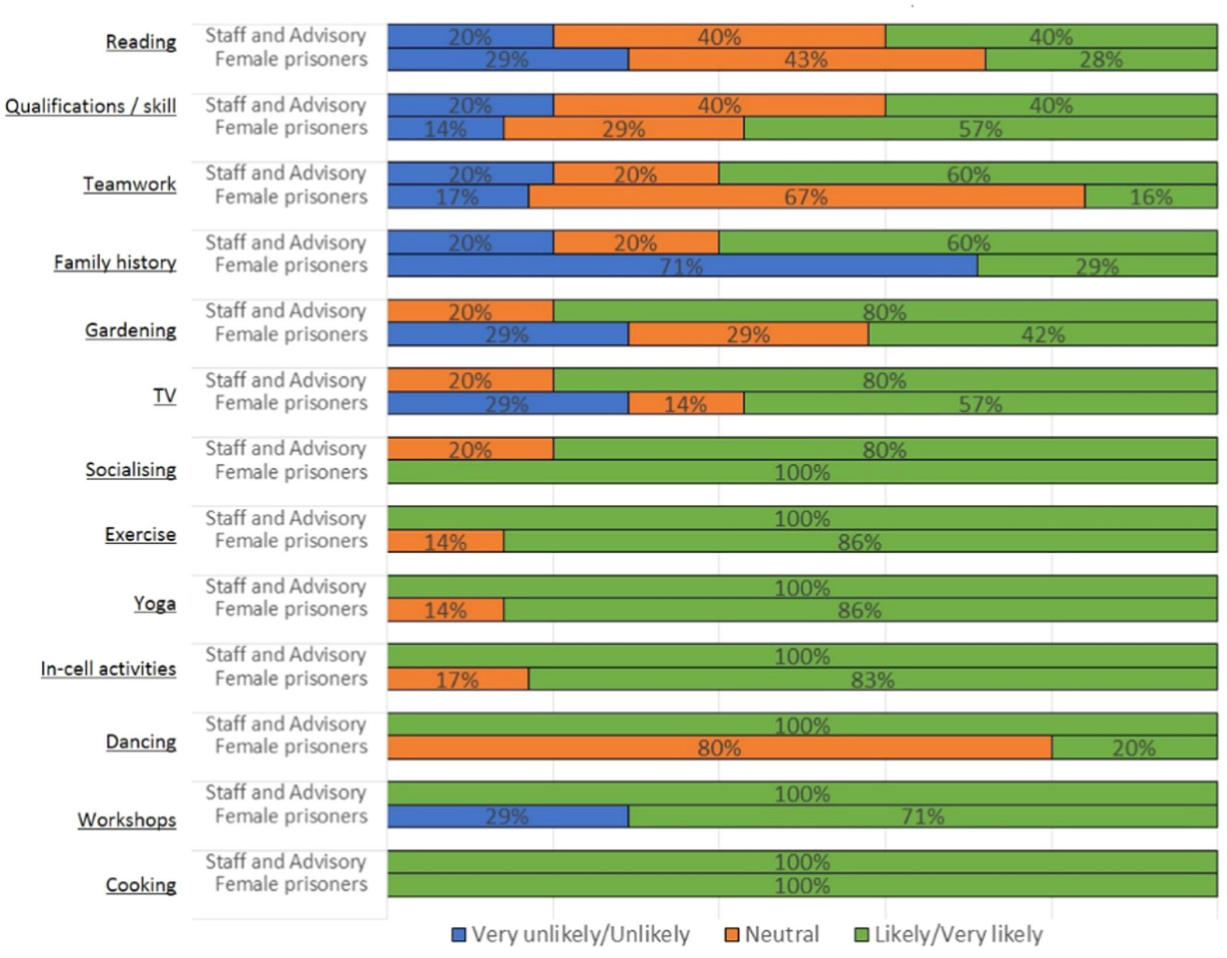
Females: acceptability—will this activity in your opinion have widespread appeal?

**Figure 5:**
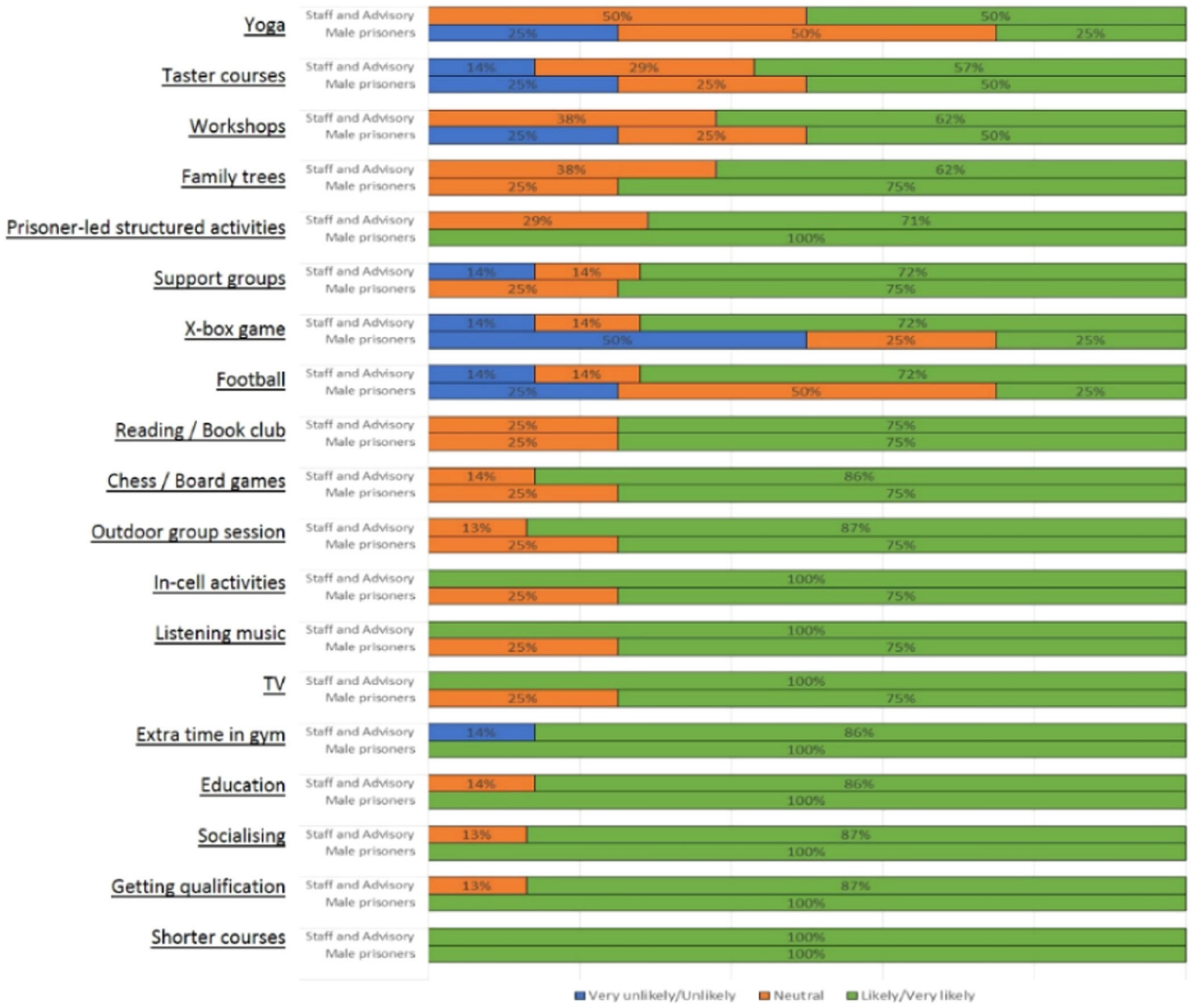
Males: acceptability—will this activity in your opinion have widespread appeal?

Views across the stakeholder groups were similar regardless of gender (Figures 4, 5). Females were more likely to report on recreational activities such as yoga, exercise, cooking and socialising in comparison to males.

Male activity preferences included chess, short courses, learning new skills and attending peer support groups (Figure 5).

##### 4.2.4.2 Activity Sustainability

The sustainability of the activities identified was similarly reported by gender. In the female prison, the sustainability of the activities ranged between 57% (cooking) and 71% (prison‐based job workshops), and 31% (workshops) and 90% (getting qualifications through education) for female prisoners and their staff. In the male prison, the sustainability of the activities ranged between 56% (getting qualification) and 88% (prison‐based job workshops), and 50% (unaccredited courses) and 84% (prison‐based job workshops), respectively.

The sustainability of leisure‐based activities ranged from 46% (family history) to 89% (socialising), and 50% (family history) to 90% (TV, reading, in‐cell activities) for female prisoners and their staff. For male prisoners and their staff, the sustainability ranged from 31% (X‐box game) to 88% (TV), and 56% (family history) to 84% (TV), respectively. Physical activities, such as exercise, gym, football, gardening and yoga, ranged from 57% (exercise) to 82% (yoga) and 65% (dancing) to 70% (gardening, yoga, exercise) for females.

#### 4.2.5 Feedback on the Workshops

Quantitative feedback on the workshops was generally positive. Overall responses on the level of interest rated as ‘not at all’, ‘not sure’ and ‘yes mostly’ or ‘yes completely’ were reported. The book club and archaeology session were most popular with all participants asking for repetition of these sessions (Table 2).

**Table 2:** Prisoner feedback using adapted feedback questionnaire.

Responses		Not at all/not really
Sites and sample	Question	Not sure
		Yes, mostly/yes completely (*n*,%)
Men only (*n* = 2)	How interesting did you find the DARS workshop? (*n* = 2: Men)	(0%)
		(1/2, 50%)
		(1/2, 50%)
	Do you think the DARS workshop should be repeated again for prisoners over 50s?	(0%)
		(0%)
		(2/2, 100%)
Women only (*n* = 6)	How interesting did you find the yoga/mindfulness workshop?	(0%)
		(1/6, 17%)
		(5/6, 83%)
	Do you think the yoga/mindfulness workshop should be repeated again for prisoners over 50s?	(0%)
		(1/6, 17%)
		(5/6, 83%)
Men and women (*n* = 8)	How interesting did you find the book club workshop? (*n* = 8: 2 men and 6 women)	(0%)
		(0%)
		(7/8, 100%: 1 missing answer)
	Do you think the book club workshop should be repeated? (*n* = 8: 2 men and 6 women)	(0%)
		(0%)
		(8/8, 100%)
	How interesting did you find the archaeology workshop? (*n* = 8: 2 men and 6 women)	(0%)
		(0%)
		(8/8, 100%)
	Do you think the archaeology workshop should be repeated? (*n* = 8: 2 men and 6 women)	(0%)
		(0%)
		(8/8, 100%z)

### 4.3 Economic Costs of the Workshop Delivery

The total cost of the intervention was estimated at £8403.44. More females than males attended the workshops with the total cost being 1.4 times higher in the female prison (£4943.66 vs. £3459.78) than compared to the male site, the average cost per participant was (£157.25 vs. £89.88), respectively. Recurrent costs were the main cost driver of the intervention (92% of the total costs). Within recurrent costs, personnel and miscellaneous items had similar costs, £3839.26 and £3788.78, respectively (Table 3).

**Table 3:** Capital and recurrent costs of PAMHOP workshops in male and female prisons, United Kingdom, 2022.

Item, recurrent costs	Male prison	Female prison	Total cost
Capital costs			
Planning and preparing the sessions			
Facilitator (£)	201.96	201.96	403.92
Healthcare staff (£)	36.60	36.60	73.20
Historian (£)	145.28	145.28	290.56
Librarian (female only) (£)	NA	7.72	7.72
Total capital (£)	383.84	391.56	775.40
Recurrent costs			
Miscellaneous			
Catering (£)	52.80	132.00	184.80
Travel (£)	81.90	287.00	368.90
Picture frames/certificates and printing (£)	440.00	1100.00	1540.00
Course fee attendance (£)	220.00	550.00	770.00
Print mindfulness script (female only) (£)	NA	1.08	1.08
Opportunity costs (£)	264.00	660.00	924.00
Personnel			
Officer (£)	358.92	249.25	608.17
Facilitator (£)	605.88	504.90	1110.78
Healthcare staff (£)	36.60	36.60	73.20
Historian (£)	1015.84	1015.84	2031.68
Librarian (female only) (£)	NA	15.43	15.43
Total recurrent (£)	3075.94	4552.10	7628.04
Grand total (capital + recurrent) (£)	3459.78	4943.66	8403.44

### 4.4 The Resulting Practical Guidance Toolkit(s)

The combined evidence from these workshops created two gendered toolkits (one male and one female). The guidance documents incorporated the survey results (Phase 2) and the findings from the workshops (Phase 3). Elements of each toolkit (https://www.york.ac.uk/healthsciences/research/mental-health/projects/physical-mental-health-older-prisoners/) include factors about how to improve common mental health in custody, how to encourage older people in custody to take part in activities and suggestions for sustainability. Three case study examples of the taster sessions are described, and partnership working with organisations and charities is listed as an example of good practice.

## 5 Discussion

To our knowledge, this is one of the first studies to utilise knowledge obtained from research evidence gaps combined with the results from a prison survey and interview data to inform the co‐production of a series of gendered workshops. We describe using the GUIDED checklist (Supporting Materials 5) [53], which recognises the importance of understanding the theory behind how and why an intervention works to [25, 54, 55, 56] maximise engagement and reduce attrition during Phase 3 of the project [22, 23, 57]. Engagement in this project was particularly important to those older people in custody who participated in this study. Evidence from the earlier Phase 1 systematic review [27] reported high dropout during treatment and interruption due to prison appointments/visits or unplanned lockdown. Staff and prisoner buy‐in [58, 59] were key to the successful delivery of the workshops. Other research recognises issues of entrenched power differences, prison bureaucracy and a lack of funding all contributed to the complexity of delivering activities in prison [58, 60, 61]. From this study, we learnt that engagement for older people in custody was about offering a choice to those that wanted to take part. Feedback from the workshop discussions suggested that those with less confidence favoured support from a peer or a member of the prison staff team to collect them from the wing when attending alone or when going to an area of the prison that they had not been to before. Attendance at the activities in this study was affected by the logistical organisation of the prison and the willingness of those in custody wanting to attend. Males in comparison to females were less likely to attend the workshops due to competing prison work and legal appointments (9 vs. 2). Release into the community during the workshop delivery occurred on a couple of occasions; brief interventions are therefore more likely to reach more people given the transient nature of some members of this population. Participants preferred having staff of a similar age group running the activities; they were keen to have their age group represented in prisoner forums and at staff meetings. Building a sense of achievement into the activities was important and easily managed with the use of high‐quality laminated certificates and the provision of refreshments. Payment for participation in our workshops was a motivating factor for some who took part.

The study examined the perceived connections between common mental *and* physical health conditions and contributed to other work that had already examined the impact of the prison environment on the mental health of males in custody [41, 43]. Our study brings new evidence documenting the mental and physical health conditions of older males *and* importantly older females in custody. The mapped connections have the potential to develop a new theoretical framework to understand the impact of custody on mental and physical health more broadly. Further research should be used to expand this consultation process with a larger and more representative sample of people in custody.

Gendered health inequalities were not as pronounced as reported in other studies [16]. Prior studies are limited by small sample sizes, which may not be representative of this population [62]. Gendered differences reported in this study focused on the disparities that were presented. Activity preferences were broad (e.g., vocational skills, short courses, and creative activities), but none were particularly focused on accessing employment through the gate. Activities were instead underpinned by having a sense of purpose, learning new things, gaining, and sharing skills and socialising with people of a similar age. The findings concur with allied recommendations from the National Institute for Health and Care Excellence (NICE). Whereby recommendations for activities of older people in community care homes include meaningful activities, that help to maintain and develop personal identify, to create a feeling of self‐control and a sense of belonging [63]. Prison site business strategies tended to focus on the needs of those that were younger; by supporting access to functional Maths and English and access to employment through the gate. Given the growing number of older people in custody, prison site strategies should be more inclusive of the needs of older people in custody.

Use of relatively inexpensive activities (e.g., a walking group around the prison grounds) and a flexible delivery model (using more than one person) helped to provide a consistent approach to the delivery of an activity. Activity delivery around the prison regime, and in different locations including the outdoor space, was favoured by participants. The prison environment clearly had a notable impact on the receipt of the activity. For example, delivery of interventions in spaces that encouraged socialisation (e.g., café mess) or were akin to space in the community (e.g., the library) was thought to be beneficial to progress promotion of trust, independence, self‐belief and empowerment in the journey of recovery.

Yoga interventions [27] in the review reported a lack of appropriate prison‐based accessible rooms to prisoners on restricted movements, resulting in limited class sizes and loss of participants due to release/lock up. In our workshop, we used the same room location for all sessions. This helped to create a consistent expectation and approach to the intervention delivery. The adapted chair yoga session notably took up less space than asking individuals to complete yoga whilst on the floor. The female prison site had access to laptops; it was suggested that such sessions and short videos could be presented via the laptops as an alternative format of in‐cell delivery, which would not require any additional space. Other barriers were challenged in the group before commencing the historical session. Some members of the group had an initial resistance to walking too far with mobility and back complaints; others anticipated that they could not draw or had come without their glasses and were therefore unable to contribute. Despite these initial concerns, all participants reported this as their favoured activity in the workshop feedback questionnaire with a series of booster sessions requested.

In the male book club and puzzle session, barriers to the delivery of the books in advance of the club were primarily due to staff shortages. Despite this, the activity session generated instead a range of creative ideas that could utilise the existing resources within the library which did not rely upon this activity happening before the session started. Examples of these sessions provide a real opportunity to increase the quality of life and improve the mental and physical health of older people in custody. Such initiatives are likely to therefore subsequently reduce the high economic and social cost of poor health upon release from prison with the use of minimal resources.

Detailed creative planning and clear partnership working showed that meaningful activities can be delivered with little additional cost. The average cost per participant for the intervention delivery was higher for females than males (£157 vs. £89) but remained low cost when compared to the costs associated with mental health and well‐being in the wider prison community. The cost difference between the male and female sites was determined by the prisoner status of a workshop participant that required a one‐to‐one staff escort in the female prison. For this reason, intervention delivery is likely to vary, and future CCAs should be used to inform the implementation of the intervention across several different prison sites. Given the nature of this work the small sample size raises concerns for the generalisability of the logic model and the need to develop the intervention. A programme of research is required to evaluate, develop and test the subsequent effectiveness using a robust research design in several male and female prison sites.

## 6 Conclusions

This work demonstrates how engagement with people in custody generates valuable research evidence to support the development of interventions for this age group. Importantly the paper highlights the relevance of Patient and Public Involvement and Engagement (PPIE) to help maximise patient benefit. Further research is required to refine the practical guidance toolkit(s) as part of recommendations for a larger programme of research to generate an evidence‐base for this population.

## Author Contributions

**Amanda E. Perry:** conceptualization, methodology, investigation, funding acquisition, project administration, supervision, validation, writing – review and editing, writing – original draft. **Thirimon Moe‐Byrne:** formal analysis, validation, writing – original draft, writing – review and editing, project administration. **John Schofield:** methodology, conceptualization, funding acquisition; data curation, writing – review and editing. **Lisa Ashton:** Methodology. **Noemia Siqueria:** methodology, data curation, formal analysis, writing – review and editing. **Sarah Knowles:** methodology, conceptualization, data curation. **Rachel Churchill:** methodology, conceptualization, funding acquisition, writing – review and editing, supervision. **Tim Colman:** data curation, funding acquisition, conceptualization, methodology. **Steve Parrott:** funding acquisition, conceptualization, methodology, supervision, writing – review and editing. **Kevin Williamson:** project administration, funding acquisition, resources.

## Disclosure

The views expressed are those of the author(s) and not necessarily those of the NIHR or the Department of Health and Social Care. The funders of the study had no role in study design, data collection, data analysis, data interpretation or writing of the report.

## Ethics Statement

Ethics were approved by the Ethics Committee (REC reference: 22/EE/0120). All study participants gave their informed consent to participate.

## Conflicts of Interest

The authors declare no conflicts of interest.

## Supporting information

Supplementary Materials.

## Data Availability

Data for this study are not available, as the participants did not agree for these to be shared publicly. The analysis code can be provided by the authors upon reasonable request.

## Endnotes

1 The Project Advisory Team consisted of representation from the women's psychological services, an ex‐prison chaplain, male psychological services, senior academic with specialist knowledge of prisons, patient public representation (one male and one female), a national specialist lead for Her Majesty's Prison and Probation Service Intervention Services.
