# Supplementary material for: Including Prisoners in Research Design: Codevelopment of a Practical Guidance Toolkit to Support Intervention Delivery to Address the Physical and Mental Health of Older Prisoners (PAMHOP) Study
Source: Health Expect. 2025 Jun 9;28(3):e70246. doi: 10.1111/hex.70246 (PMC12147402; doi:10.1111/hex.70246)
Supplement: Supplementary file 1 — Supplementary Materials. [file HEX-28-e70246-s001.docx]

Supplementary materials 1 Theory of Change causal Model

Source: The effect of a peer-led problem-support mentor intervention on self-harm and violence in prison: An interrupted time series analysis using routinely collected prison data. Perry, Amanda E. et al. eClinicalMedicine, Volume 32, 100702.


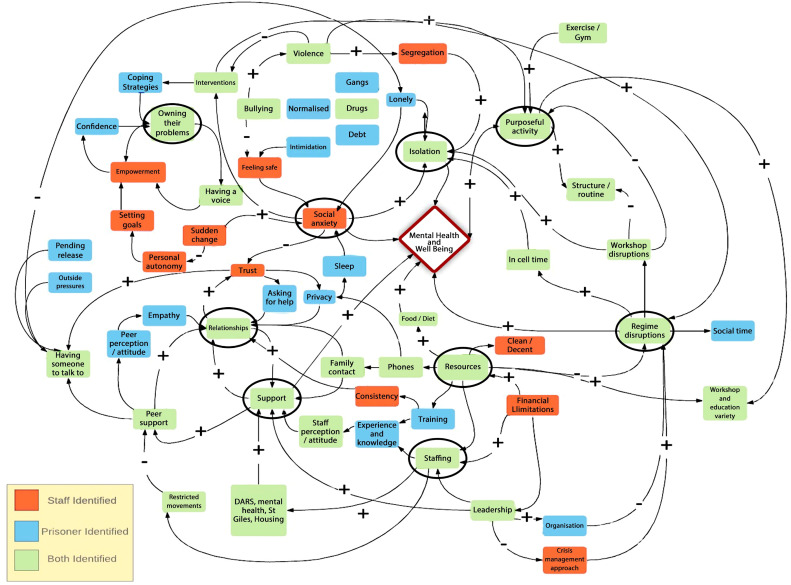


Supplementary 2: Initial logic model

**Inputs**

**Impacts**

**Outcomes**

**Activities**

*Workshops* will address if activity is:

Feasible to deliver in the space of the prison (can people in custody be taken to it, is there sufficient room or quiet space)

Acceptable to the people in custody: achieves Outcomes identified as important to them and is perceived as personalised

Tailored and or adapted activities by task and or duration.

Materials/content tailored to the population in terms of age and physical co-morbidity, and potential for personalisation (e.g. working in group or working alone) *(from Review and Qualitative*)

Identification and support to access for target population.

Encourage engagement through peer-led support.

**Individual/Intervention Level**

Longer term individual impacts to be agreed through Stakeholder Consultation

For assessing fit within context (*from Review and Qualitative*).

Drop-out/number of sessions attended, including interrupted attendance due to movement or restriction, duration, and intensity.

Reach (to different population subgroups). Ability to deliver core ingredients as intended.

For people in custody (*from Qualitative and Survey*)

Socialising, reducing boredom, occupying time.

Physical aspect (crafting for women, gym for men)

Sense of progress or achievement

Developing skills, Sense of purpose

Improved self-esteem, confidence, mental health, and well-being

Improved contact with family members

**Organisational Level**

Legacy resources or integration with existing providers to continue provision (from Qualitative and Survey)

Clear protocol for staff to support access e.g. timing of sessions (*from Qualitative and Stakeholder Consultation*)

Delivery in different spaces (e.g., in cell, outdoors, in other rooms such as the library).

Staff buy-in at senior level and adequate and consistent allocation of staff to support access/movement between sessions (*from Review and Qualitative)*

Staff working together in small teams across different disciplines.

Development of an over 50 strategies.

Suitably trained staff of an appropriate age.

**Evaluation Outcome**

Feasibility & Acceptability

Sustainability &Penetration

Cost & Fidelity

Appropriateness &Adoption

Supplementary 3: Case study persona

**Imagine you are Anjuman/Patrick**

| Anjuman was recalled into prison on licence for not following the rules. On the night before she was arrested, she was involved in a car incident in which her car was stolen. She doesn’t know what has happened to her car and she is still paying road tax. She needs her car on the outside because she uses it for her job as a Care Worker. Anjuman was originally born in Poland and has been in the UK for five years. Her understanding of English is not great. Anjuman enjoys her work as a care worker and has done it for many years before coming to the UK. She is worried that when she is released she won’t be able to work and she won’t have any money. She finds it hard to get on with the younger people in custody; and is apprehensive about trying to ask staff questions about how to resolve her problem. She is now retired in the prison and doesn’t have a job. She has low self-esteem and confidence and is becoming more isolated in her cell. In the past she has suffered from bouts of depression and is overweight. |
| --- |

**Imagine you are Sofia/Joseph**

Sofia, is an older woman, who worked in the banking industry for 25 years. She was finally convicted for fraud after a lengthy case and Sofia was sent to prison for 9 years. She was shocked by her incarceration and was transferred from one prison to another very quickly. She is struggling to adjust to prison life. She feels completely out of place, and she looks like she is not looking after herself. She has pain in her back; experienced from an old motorbike injury. Her son comes to visit her– but he is ashamed of his Mother’s behaviour and tells her he does not want to be part of her life anymore. Sofia feels isolated, angry and lonely. Sofia refuses to come out of her cell when asked to by a member of staff; she feels unsafe and intimidated. Her mental health is starting to suffer but she doesn’t want to talk about it. A member of staff identifies Sofia as needing some help.

**Imagine you are Nadia/Andrew**

| Nadia has a long history of self-harm behaviour and she has been monitored by staff for her self-harm behaviour for many months; she is also a diabetic and needs to watch what she eats. She has harmed herself ever since being in prison and has committed quite a high-profile crime which restricts her from moving around the prison; she is often in conflict with staff. Nadia is quite quiet and generally keeps herself to herself – she has a grown-up family on the outside. They haven’t been able to pay the mortgage on the house for two months and the families’ savings have all run out. Last week her husband came to visit upset, saying that he was worried that the house would be repossessed. She feels completely out of control, she is panicking about what she can do to help support her family and her husband. She doesn’t have a clear plan in her head about how she can help to resolve the situation and is feeling very anxious and isn’t sleeping well. She feels like she needs to advise her husband on what they should do, she doesn’t know where to start and she thinks that time is running out for them. She blames herself for the situation and has been more reluctant to come out of her cell and go to work. She doesn’t like doing new jobs or activities in the prison and is apprehensive about trying something new; she experiences social stigma from having to be escorted around the prison separately. |
| --- |

Barriers to engagement Mental health Physical health

Supplementary 4: Adapted feedback questionnaire

**PAMHOP Workshops Feedback Questionnaire – Female version**

Study site: Participant’s ID

For each statement please **PUT A CIRCLE** round the option that best matches your view or leave comments.

1) Have you been involved in research before?

**Yes / No (please circle).**

If yes, please give details

2a) How interesting did you find the Archaeology/historical workshop?

Not at all Not really Not sure Yes, mostly Yes,completely

2b) Do you think the Archaeology/historical workshop should be repeated again for people in custody over 50s?

Not at all Not really Not sure Yes, mostly Yes,completely

3a) How interesting did you find the Yoga/Mindfulness workshop?

Not at all Not really Not sure Yes, mostly Yes,completely

3b) Do you think the yoga/mindfulness workshop should be repeated again for people in custody over 50s?

Not at all Not really Not sure Yes, mostly Yes,completely

4a) How interesting did you find the book club/crafts workshop?

Not at all Not really Not sure Yes, mostly Yes,completely

4b) Do you think the book club/crafts workshop should be repeated again for people in custody over 50s?

Not at all Not really Not sure Yes, mostly Yes,completely

5) Did you experience any benefit to your mental health?

Not at all Not really Not sure Yes, mostly Yes,completely

6) Did you experience any benefit to your physical health?

Not at all Not really Not sure Yes, mostly Yes,completely

7) If you did get some benefits to your mental/physical health, what were they?

8) Did you experience any problems or barriers that made you less likely to want to attend?

9) Was there anything you wanted to know or add but which wasn’t included in the workshops?

**Yes / No (please circle).** If yes, please write them here:

**Thank you for taking the time to complete this questionnaire.**

Supplementary 4: Adapted feedback questionnaire

**PAMHOP Workshops Feedback Questionnaire – Male version**

Study site: Participant’s ID

For each statement please **PUT A CIRCLE** round the option that best matches your view or leave comments.

1) Have you been involved in research before?

**Yes / No (please circle).** If yes, please give details

2a) How interesting did you find the Archaeology/historical workshop?

Not at all Not really Not sure Yes, mostly Yes,completely

2b) Do you think the Archaeology/historical workshop should be repeated again for people in custody over 50s?

Not at all Not really Not sure Yes, mostly Yes,completely

3a) How interesting did you find the DARS workshop?

Not at all Not really Not sure Yes, mostly Yes,completely

3b) Do you think the DARS workshop should be repeated again for people in custody over 50s?

Not at all Not really Not sure Yes, mostly Yes,completely

4a) How interesting did you find the book club/games workshop?

Not at all Not really Not sure Yes, mostly Yes,completely

4b) Do you think the book club/games workshop should be repeated again for people in custody over 50s?

Not at all Not really Not sure Yes, mostly Yes,completely

5) Did you experience any benefit to your mental health?

Not at all Not really Not sure Yes, mostly Yes,completely

6) Did you experience any benefit to your physical health?

Not at all Not really Not sure Yes, mostly Yes,completely

7) If you did get some benefits to your mental/physical health what were they?

8) Did you experience any problems or barriers that made you less likely to want to attend?

9) Was there anything you wanted to know or add but which wasn’t included in the workshops?

**Yes / No (please circle).** If yes, please write them here:

**Thank you for taking the time to complete this questionnaire.**

**Supplementary 5 -GUIDED Checklist**

| **Item description** | **Explanation** | **Page in manuscript where item is located** | **Other*** |
| --- | --- | --- | --- |
| 1. Report the context for which the intervention was developed. | Understanding the context in which an intervention was developed informs readers about the suitability and transferability of the intervention to the context in which they are considering evaluating, adapting or using the intervention. Context here can include place, organisational and wider sociopolitical factors that may influence the development and/or delivery of the intervention (15). | Pg 4,5 |  |
| 2. Report the purpose of the intervention development process. | Clearly describing the purpose of the intervention specifies what it sets out to achieve. The purpose may be informed by research priorities, for example those identified in systematic reviews, evidence gaps set out in practice guidance such as The National Institute for Health and Care Excellence or specific prioritisation exercises such as those undertaken with patients and practitioners through the James Lind Alliance. | Pg 4,5 |  |
| 3. Report the target population for the intervention development process. | The target population is the population that will potentially benefit from the intervention – this may include patients, clinicians, and/or members of the public. If the target population is clearly described then readers will be able to understand the relevance of the intervention to their own research or practice. Health inequalities, gender and ethnicity are features of the target population that may be relevant to intervention development processes. | Pg 5 |  |
| 4. Report how any published intervention development approach contributed to the development process | Many formal intervention development approaches exist and are used to guide the intervention development process (e.g. 6Squid (16) or The Person Based Approach to Intervention Development (17)). Where a formal intervention development approach is used, it is helpful to describe the process that was followed, including any deviations. More general approaches to intervention development also exist and have been categorised as follows (3):- Target Population-centred intervention development; evidence and theory-based intervention development; partnership intervention development; implementation-based intervention development; efficacy based intervention development; step or phased-based intervention development; and intervention-specific intervention development (3). These approaches do not always have specific guidance that describe their use. Nevertheless, it is helpful to give a rich description of how any published approach was operationalised | Pg 6-11 |  |
| 5. Report how evidence from different sources informed the intervention development process. | Intervention development is often based on published evidence and/or primary data that has been collected to inform the intervention development process. It is useful to describe and reference all forms of evidence and data that have informed the development of the intervention because evidence bases can change rapidly, and to explain the manner in which the evidence and/or data was used. Understanding what evidence was and was not available at the time of intervention development can help readers to assess transferability to their current situation. | Pg 4-6 |  |
| 6. Report how/if published theory informed the intervention development process. | Reporting whether and how theory informed the intervention development process aids the reader’s understanding of the theoretical rationale that underpins the intervention. Though not mentioned in the e-Delphi or consensus meeting, it became increasingly apparent through the development of our guidance that this theory item could relate to either existing published theory or programme theory | Pg 6,7, Figure 1, supp material 1 |  |
| 7. Report any use of components from an existing intervention in the current intervention development process. | Some interventions are developed with components that have been adopted from existing interventions. Clearly identifying components that have been adopted or adapted and acknowledging their original source helps the reader to understand and distinguish between the novel and adopted components of the new intervention. | Pg 4-6 |  |
| 8. Report any guiding principles, people or factors that were prioritised when making decisions during the intervention development process. | Reporting any guiding principles that governed the development of the application helps the reader to understand the authors’ reasoning behind the decisions that were made. These could include the examples of particular populations who views are being considered when designing the intervention, the modality that is viewed as being most appropriate, design features considered important for the target population, or the potential for the intervention to be scaled up. | Pg 6-9 |  |
| 9. Report how stakeholders contributed to the intervention development process. | Potential stakeholders can include patient and community representatives, local and national policy makers, health care providers and those paying for or commissioning health care. Each of these groups may influence the intervention development process in different ways. Specifying how differing groups of stakeholders contributed to the intervention development process helps the reader to understand how stakeholders were involved and the degree of influence they had on the overall process. Further detail on how to integrate stakeholder contributions within intervention reporting are available (19). | Pg 5-10 |  |
| 10. Report how the intervention changed in content and format from the start of the intervention development process. | Intervention development is frequently an iterative process. The conclusion of the initial phase of intervention development does not necessarily mean that all uncertainties have been addressed. It is helpful to list remaining uncertainties such as the intervention intensity, mode of delivery, materials, procedures, or type of location that the intervention is most suitable for. This can guide other researchers to potential future areas of research and practitioners about uncertainties relevant to their healthcare context. | NA |  |
| 11. Report any changes to interventions required or likely to be required for subgroups. | Specifying any changes that the intervention development team perceive are required for the intervention to be delivered or tailored to specific subgroups enables readers to understand the applicability of the intervention to their target population or context. These changes could include changes to personnel delivering the intervention, to the content of the intervention, or to the mode of delivery of the intervention. | NA |  |
| 12. Report important uncertainties at the end of the intervention development process. | Intervention development is frequently an iterative process. The conclusion of the initial phase of intervention development does not necessarily mean that all uncertainties have been addressed. It is helpful to list remaining uncertainties such as the intervention intensity, mode of delivery, materials, procedures, or type of location that the intervention is most suitable for. This can guide other researchers to potential future areas of research and practitioners about uncertainties relevant to their healthcare context. | Pg 17-19 |  |
| 13. Follow TIDieR guidance when describing the developed intervention. | Interventions have been poorly reported for a number of years. In response to this, internationally recognized guidance has been published to support the high quality reporting of health care? interventions^5^and public health interventions^14^. This guidance should therefore be followed when describing a developed intervention. | NA |  |
| 14. Report the intervention development process in an open access format. | Unless reports of intervention development are available people considering using an intervention cannot understand the process that was undertaken and make a judgement about its appropriateness to their context. It also limits cumulative learning about intervention development methodology and observed consequences at later evaluation, translation and implementation stages. Reporting intervention development in an open access (Gold or Green) publishing format increases the accessibility and visibility of intervention development research and makes it more likely to be read and used. Potential platforms for open access publication of intervention development include open access journal publications, freely accessible funder reports or a study webpage that details the intervention development process. | NA |  |
